# Stage-specific embryonic antigen-4 is a histological marker reflecting the malignant behavior of prostate cancer

**DOI:** 10.1007/s10719-019-09882-2

**Published:** 2019-06-26

**Authors:** Yuichiro Nakamura, Yasuyoshi Miyata, Tomohiro Matsuo, Yohei Shida, Tomoaki Hakariya, Kojiro Ohba, Takenobu Taima, Akihiro Ito, Tetsuji Suda, Sen-itiroh Hakomori, Seiichi Saito, Hideki Sakai

**Affiliations:** 1 Department of Urology, Nagasaki University Graduate School of Biomedical Sciences, 1-7-1 Sakamoto, Nagasaki, 852-8501 Japan; 2 Department of Urology, Tohoku University Graduate School of Medicine, Miyagi, 980-8574 Japan; 3 Department of Urology, University of the Ryukyus, Okinawa, 903-0215 Japan; 4 Departments of Pathobiology and Global Health, University of Washington, Seattle, WA 98112 USA

**Keywords:** SSEA-4, Tumor-infiltrating immune cells, Biochemical recurrence, Apoptosis · prostate cancer

## Abstract

Stage-specific embryonic antigen-4 (SSEA-4), a specific marker for pluripotent stem cells, plays an important role in the malignant behavior of several cancers. Here, SSEA-4 expression was evaluated by immunohistochemistry using monoclonal antibody RM1 specific to SSEA-4 in 181 and 117 prostate cancer (PC) specimens obtained by biopsy and radical prostatectomy (RP), respectively. The relationships between SSEA-4 expression in cancer cells or the presence of SSEA-4-positive tumor-infiltrating immune cells (TICs) and clinicopathological parameters were analyzed. SSEA-4 expression in cancer cells was significantly associated with Gleason score, local progression, and lymph node and distant metastasis. In RP specimens, high SSEA-4 expression in cancer cells and the presence of SSEA-4-positive TICs were significant predictors of pT3, *i.e*., invasion and worse biochemical recurrence (BCR) after RP, respectively, in univariate analysis. In contrast, combination of high SSEA-4 expression in cancer cells and the presence of SSEA-4-positive TICs was an independent predictor for pT3 and BCR in multivariate analysis. Biologically this combination was also independently associated with suppression of apoptosis. Thus, the co-expression of SSEA-4 in cancer cells and TICs may have crucial roles in the malignant aggressiveness and prognosis of PC. Invasive potential and suppression of apoptosis may be linked to SSEA-4 expression.

## Introduction

Solid tumors are composed of heterogeneous cell populations with different biological and molecular characteristics. Among heterogeneous cell populations, cancer stem cells (CSCs) play crucial roles in tumor growth and chemo-resistance in various types of malignancies [1, 2]. SSEA-4, a sialic acid-containing glycolipid, is recognized as a specific marker for pluripotent stem cells, and is reportedly a useful marker for the detection of CSCs [3]. Furthermore, SSEA-4 expression has been associated with the remodeling of gastrointestinal cancer cell lines including those in esophageal, stomach, colorectal, liver, pancreatic, and cholangiocellular cancer [4, 5]. In addition, SSEA-4 expression is positively associated with malignant behavior in breast cancer [6, 7] and glioblastoma [8]. Based on these facts, it is speculated that SSEA-4 expression in cancer cells plays a significant role in the malignant aggressiveness of prostate cancer (PC).

Mesenchymal stem cells (MSCs) and tumor-infiltrating immune cells (TICs) that express stem cell markers are associated with the regulation of cancer-related molecules and characteristics in various cancers including PC [9–11]. Interestingly, in breast cancer, SSEA-4 expression was detected not only in cancer cells, but also cancer stem cells [12]. In addition, it was reported that the presence of SSEA-4-positive MSCs in PC tissues was significantly associated with disease-free survival and overall survival in PC patients [10]. However, no study has focused on the pathological significance and prognostic role of SSEA-4 expression in PC cells. Combined expression pattern of SSEA-4 in cancer cells and the presence of SSEA-4-positive TICs in PC have not been studied. Furthermore, the previous researches on SSEA-4 expression in PC used commercially available monoclonal antibody (mAb) MC813–70, which is also reactive to GM1b and GD1a in addition to SSEA-4 [3]. Therefore, we investigated the role of SSEA-4 expression in cancer cells and TICs in relation to the malignant aggressiveness in PC using mAb RM1, which is specific to SSEA-4 and does not react to GM1b or GD1a [13, 14].

## Materials and methods

### Patients

SSEA-4 expression in cancer cells was evaluated in biopsy samples from 181 PC cases and 117 radical prostatectomy (RP) specimens obtained from the Nagasaki University Hospital. In the RP cases, patients with pT4, metastasis, and/or peri-operative treatments including neo-adjuvant hormonal therapy were excluded. As non-tumoral controls, 60 specimens obtained by trans-urethral resection of benign hyperplasia were also evaluated. The study protocol was in accordance with the standards upheld by the Ethics Committee of Nagasaki University Hospital and with those of the 1964 Helsinki Declaration and its later amendments for ethical research involving human subjects, and written informed consent was obtained from all the individual participants included in this study.

### Immunohistochemistry

We used a mouse IgM monoclonal antibody, RM1, that was established as a specific mAb targeting SSEA-4 [13]. The specificity of this antibody in immunohistochemistry has been confirmed in previous studies [14, 15]. In addition, we used an anti-Ki-67 antibody (Dako Corp., Glostrup, Denmark) to evaluate cell proliferation. Furthermore, *in situ* labeling for apoptosis was also performed for apoptotic cell detection. Immunohistochemical staining and terminal transferase dUTP nick end labeling (TUNEL) assays were performed according to our previous reports [16–18]. Regrading SSEA-4, antigen retrieval was performed at 95 °C for 40 min in 0.01 M sodium citrate buffer (pH 9.0). The sections were incubated overnight with primary antibodies at 4 °C, and then incubated with peroxidase using the CSA II kit for SSEA-4. Various specimens, such as renal cell carcinoma and tonsil specimens, in which immunoreactivity to the relevant antigens was confirmed in preliminary studies, were used as positive controls for SSEA-4 and Ki-67, respectively. For the TUNEL method, the ApopTag *In Situ* Apoptosis Detection Kit (Intergen Company, Purchase, NY) was used, and positive and negative control sections were prepared as described by the manufacturer.

### Evaluation of immunohistochemistry and TUNEL

The SSEA-4 expression was assessed semi-quantitatively, taking into account the staining intensity and percentage of positively stained cancer cells in 200 high-power fields (HPFs). Briefly, the SSEA-4 expression was graded as ‘none’, ‘weak’, ‘moderate’, and ‘strong’, and then, it was finally scored using the following scale: 0, no staining; 1, weak and/or focal staining (<10% of cells); 2, moderate or strong staining (10–50% of cells); and 3, moderate or strong staining (>50% of cells) according to previous studies [8, 19, 20]. Finally, scores of 2 and 3 were judged as denoting high SSEA-4 expression in PC cells. TICs were examined at 5–10 hot-spot HPFs within the tumor areas, and the SSEA-4 expression in the TICs was categorized as absent or present according to previous reports [10]. The proliferative index (PI) represents the percentage of Ki-67-positive cells. The apoptotic index (AI) was estimated by the percentage of TUNEL-positive cells. These semi-quantitative analyses were independently performed by three investigators (Y.N., T.M., and Y.M.) who were blinded to the patients’ clinical features and survival data. The presence of SSEA-4-positive TICs was judged in accordance with the majority decision principle. All the slides were examined using a Nikon E-400 microscope, and digital images were captured (Nikon DU100, Japan). Furthermore, we used a computer-aided image analysis system (Win ROOF, version 5.0, MITANI Corp., Japan) to calculate the statistical variables.

### Statistical analyses

Data were expressed as means ± standard deviation or median / interquartile range. Student’s t test or a Mann-Whitney U test was used to compare continuous variables. Scheffé’s method was used for multiple data comparisons. Survival analyses were performed using Kaplan-Meier survival curves and log rank *P* values. In addition, Cox proportional hazards model was used, and the results were described as hazard ratios (HRs) with their 95% confidence intervals (CIs) and P values. All the statistical analyses were two-sided, and significance was set at *P* < 0.05. Finally, all statistical analyses were performed on a personal computer with the statistical package StatView for Windows (version 5.0, Abacus Concept, Inc., Berkeley, CA).

## Results

### SSEA-4 expression

**Fig. 1:**
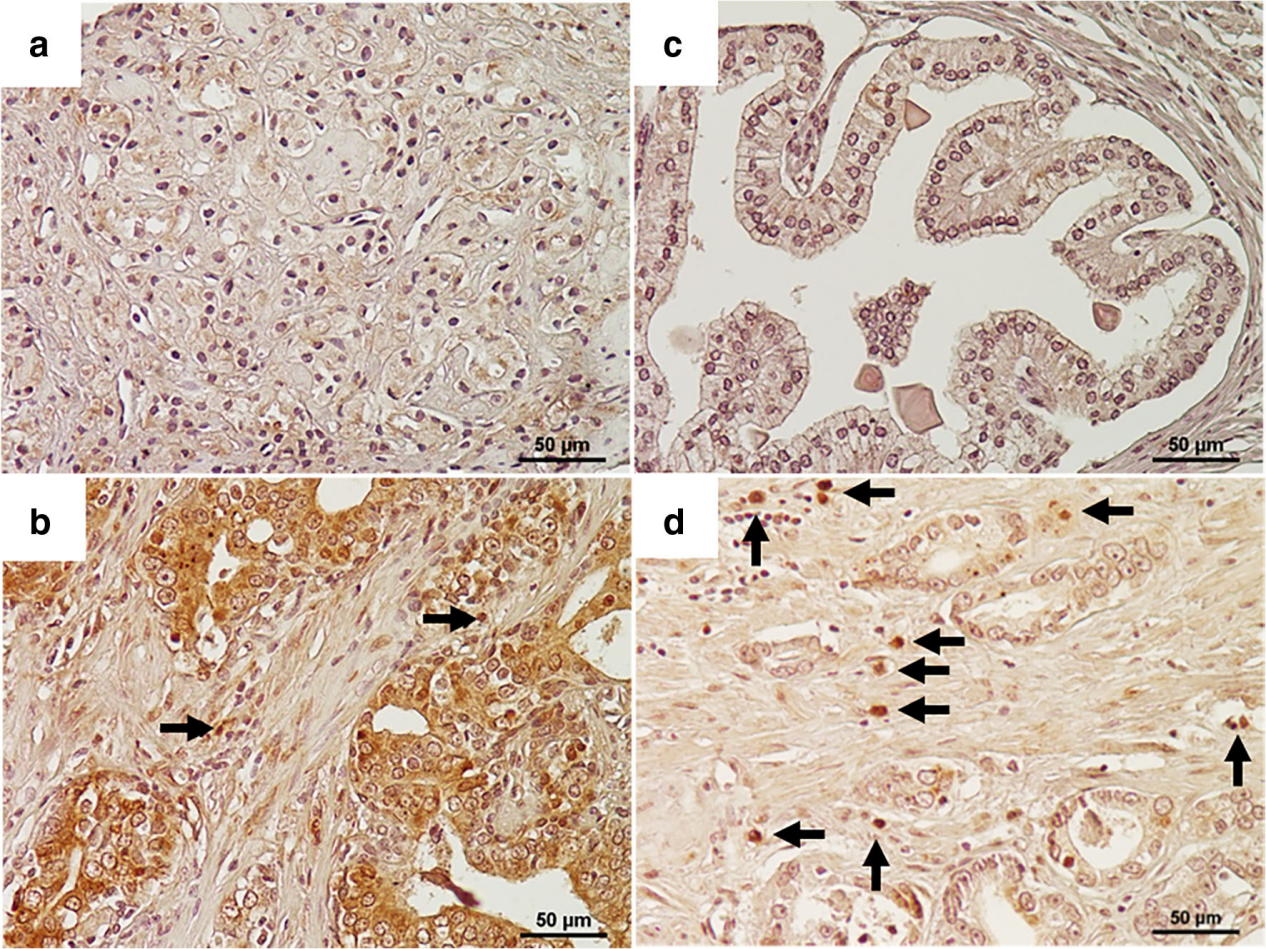
Representative examples of SSEA-4 expression in human prostate cancer tissues. With regard to prostate cancer cells, representative examples of low and high expression are shown in Figs. 1a, b, respectively. As shown in Fig. 1c, almost all the glands in the non-tumoral tissues showed weak or no expression. Representative examples of SSEA-4-positive TICs in prostate cancer tissues are shown in Fig. 1d. Arrows means SSEA-4-positive TICs. (Magnificence in all figures: X 400). SSEA, stage-specific embryonic antigen; TIC, tumor-infiltrating immune cells

SSEA-4 expression was mainly detected in the cytoplasm and a part of the cell membrane in cancer cells, and representative examples of low and high SSEA-4 expression in cancer cells are shown in Fig. 1a, b, respectively. In the non-tumoral tissues, almost all the glands showed weak or no expression (Fig. 1c). The presence of SSEA-4-positive TICs in PC tissues is shown in Fig. 1d. Finally, of the 60 non-tumoral specimens, only three (6.7%) were judged as having high SSEA-4 expression. On the other hand, we noticed no specific staining pattern or distribution in these 3 non-tumoral specimens. Of the biopsy specimens obtained from the PC patients, 57 (31.5%) ones were judged as having high SSEA-4 expression. Thus, the percentage of tissues with high SSEA-4 expression in the PC cells was remarkably (*P* < 0.001) higher that of the non-tumoral tissues. With regard to TICs, SSEA-4-positive TICs were detected in four of 60 (6.7%) of the non-tumoral tissues and 27 of 117 (23.1%) of the RP specimens; this difference reached significance (*P* < 0.001).

### Correlation with pathological features

In the 181 biopsy specimens, positive relationships were observed between SSEA-4 expression in the PC cells, as well as the Gleason score (GS) and T, N, and M stages (*P* < 0.001) (Table 1). Next, similar analyses were performed in the RP specimens as the clinical T stage evaluated by imaging examinations and the GS in biopsy specimens do not always accurately reflect pathological features. As shown in Table 2, these analyses also showed the presence of positive relationships between SSEA-4 expression in cancer cells and the GS (*P* < 0.001) and pT stages (*P* = 0.012). Furthermore, the frequency of the presence of SSEA-4-positive TICs in pT3 disease was significantly higher (*P* = 0.037) than that in pT2 disease; however, such significant differences were not observed in the GS (*P* = 0.233; Table 2). The frequency of combination of high SSEA-4 expression in cancer cells and that of SSEA-4-positive TICs in the high GS (21.6%) were remarkably higher (*P* < 0.001) than in the low GS cases (2.7%; Table 2). Similarly, combination of SSEA-4 expression in cancer cells and the presence of SSEA-4-positive TICs was significantly correlated with pT stage (*P* = 0.010, Table 2).

**Table 1:** SSEA-4 expression in cancer cells and clinicopathological features in 181 biopsy specimens

Variables	No. Pts	SSEA-4 expression		*P* value
		Low: *n* = 124	High: *n* = 57	
Serum PSA, median / IQR	181	13.1 / 8.1–34.0	64.0 / 19.4–185.0	< 0.001
Gleason score, n (%)				< 0.001
Low; < 7	46	38 (82.6)	8 (17.4)	
Middle; 7	53	42 (79.2)	11 (20.8)	
High; > 7	82	44 (53.7)	38 (46.3)	
Clinical T stage				< 0.001
T1	37	32 (86.5)	5 (13.5)	
T2	65	54 (83.1)	11 (16.9)	
T3	63	35 (55.6)	28 (44.4)	
T4	16	3 (18.8)	13 (81.3)	
Clinical N stage				< 0.001
N0	158	119 (75.3)	39 (24.7)	
N1	23	5 (21.7)	18 (78.3)	
Clinical M stage				< 0.001
M0	147	114 (77.6)	33 (22.4)	
M1	34	10 (29.4)	23 (70.6)	

Pts patients, IQR interquartile range, RP radical prostatectomy

**Table 2:** SSEA-4-related parameters and clinicopathological features in radical prostatectomy specimens

Variables	No Pts	SSEA-4 expression in cancer cells			*P* value
		Low; *n* = 85	High; *n* = 32		
Age; mean / SD		63.2 / 6.5	65.0 / 5.3		0.147
Serum PSA level; ng/ml		10.2 / 8.3	11.7 / 7.99		0.395
Gleason score					< 0.001
Low; < 7	37	34 (91.9)	3 (8.1)		
Middle; 7	43	34 (79.1)	9 (20.9)		
High; > 7	37	17 (45.9)	20 (54.1)		
pT stage					0.012
pT2	76	61 (80.3)	15 (19.7)		
pT3	41	24 (58.5)	17 (41.5)		
		SSEA-4-positive TICs			
		Absence; *N* = 90	Presence; *N* = 27		
Age; mean / SD		63.7 / 6.3	63.4 / 5.9		0.806
Serum PSA level		10.5 / 8.2	11.2 / 8.4		0.679
Gleason score					0.233
Low; < 7	37	31 (83.8)	6 (16.2)		
Middle; 7	43	34 (79.1)	9 (20.9)		
High; > 7	37	25 (67.6)	12 (32.4)		
pT stage					0.037
pT2	76	63 (82.9)	13 (17.2)		
pT3	41	27 (65.9)	14 (34.1)		
		Pattern of cancer cell expression / TICs			
		Low / absence	High or presence	High / presence	
Age; mean / SD		63.1 / 6.5	64.9 / 6.0	63.7 / 5.3	NS
Serum PSA level		9.5 / 5.8	12.7 / 12.3	11.7 / 7.5	NS
Gleason score					< 0.001
Low; < 7	37	29 (78.4)	7 (18.9)	1 (2.7)	
Middle; 7	43	31 (72.1)	6 (14.0)	6 (14.0)	
High; > 7	37	12 (32.4)	17 (45.9)	8 (21.6)	
pT stage					0.010
pT2	76	53 (69.7)	18 (23.7)	5 (6.6)	
pT3	41	19 (46.3)	12 (29.3)	10 (24.4)	

Pts patients, PSA prostate specific antigen, TICs tumor infiltrating immune cells, NS not significance

Based on these results, we investigated the pathological significance of such SSEA-4-related parameters in cancer cell invasion (pT3) in RP specimens using a multivariate analysis model including the GS (Table 3). In univariate logistic regression analysis, either of the two SSEA-4-related parameters and combination of both were significantly associated with tumor invasion (pT3) (Table 3), respectively. In multivariate analysis models the combination pattern of the high expression of SSEA-4 in cancer cells and the presence of SSEA-4-positive TICs was an independent predictor of pT3 stage (OR = 4.48; 95% CI = 1.29–15.52; *P* = 0.018, Table 3). However, neither one of SSEA-4-related parameters was an independent predictor of pT3 stage (Table 3).

**Table 3:** Influence of SSEA-4 expression for invasion (pT3) and biochemical recurrence

Variables	For invasion (pT3)			For BCR		
	OR	95% CI	*P* value	HR	95% CI	*P* value
Uni-variate analyses						
SSEA-4 in cancer cells:						
High	2.88	1.25–6.67	0.014	2.86	1.43–5.74	0.003
SSEA-4-positive TICs:						
Presence	2.51	1.04–6.05	0.040	2.57	1.25–5.27	0.010
Pattern of cancer cells and TICs:						
One of them: high or presence	1.86	0.76–4.57	0.176	2.79	1.24–6.22	0.013
High and presence	5.58	1.69–18.43	0.005	5.76	2.30–14.38	< 0.001
Multi-variate analyses						
For SSEA-4 in cancer cells						
pT stage: T3	–	–	–	3.97	1.86–8.46	< 0.001
Gleason score: Middle	1.93	0.70–5.33	0.202	1.33	0.53–3.36	0.547
High	2.10	0.69–6.37	0.190	1.64	0.63–7.25	0.310
Cancer cell: High	2.33	0.93–5.86	0.073	1.90	0.90–4.01	0.095
For SSEA-4-positive TICs						
pT stage: T3	–	–	–	3.98	1.86–8.52	< 0.001
Gleason score: Middle	2.10	0.76–5.76	0.150	1.39	0.55–3.54	0.491
High	2.76	0.98–7.76	0.054	1.95	0.77–4.97	0.161
SSEA-4-positive TICs: Presence	2.27	0.92–5.59	0.075	1.76	0.83–3.74	0.138
For pattern of cancer cells and TICs						
pT stage: T3	–	–	–	3.47	1.58–7.64	0.002
Gleason score: Middle	1.87	0.67–5.22	0.231	1.50	0.59–3.82	0.399
High	2.08	0.69–6.25	0.193	1.62	0.63–4.17	0.318
Pattern: High or presence	1.63	0.61–4.33	0.330	2.25	0.95–5.30	0.064
High and presence	4.48	1.29–15.52	0.018	2.89	1.05–7.96	0.040

OR odds ratio, HR hazard ratio, CI confidential intervals, TICs tumor-infiltrating immune cells, GS Gleason score

### Correlation with biochemical recurrence

**Fig. 2:**
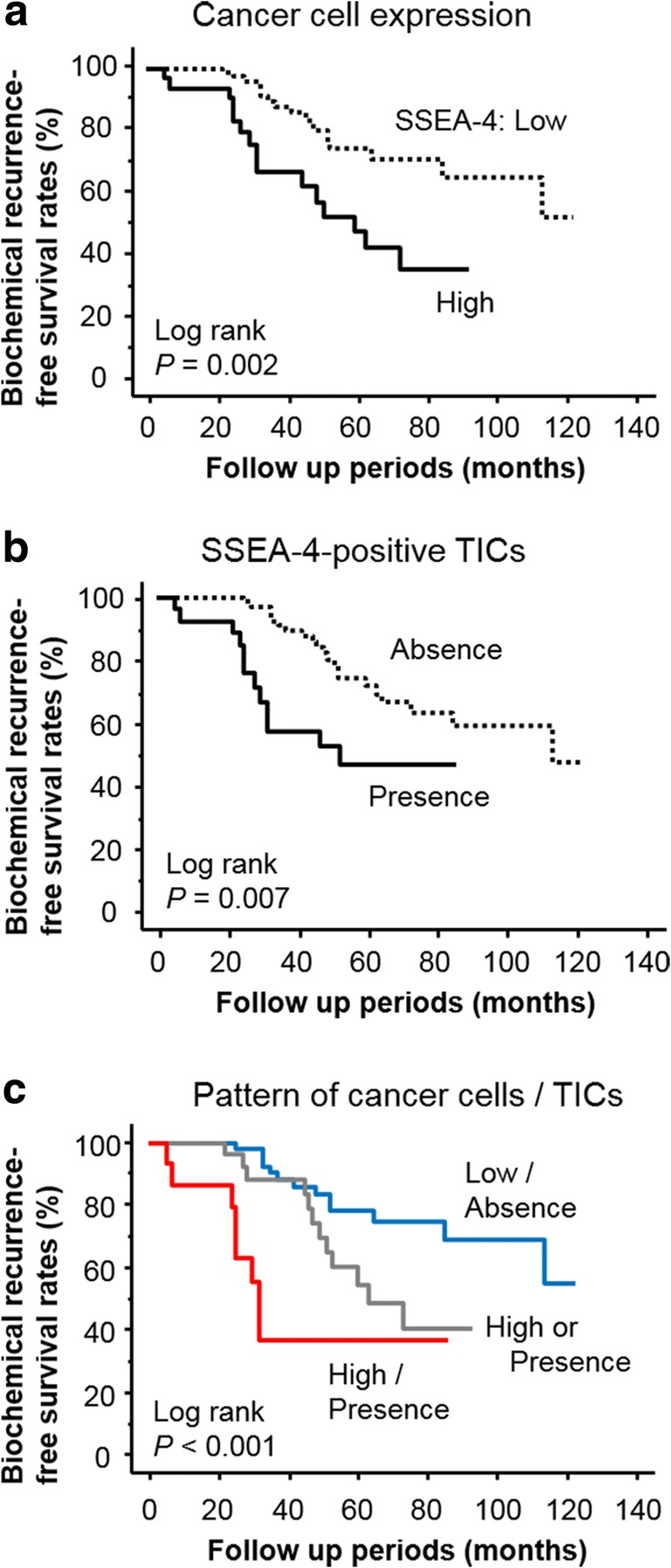
Kaplan-Meier survival curves for biochemical recurrence-free survival. All parameters of SSEA-4 expression in cancer cells (**a**), SSEA-4-positive TICs (**b**), and their combined patterns (**c**) were significantly associated with BCR in prostate cancer patients treated with radical prostatectomy. BCR, biochemical recurrence; SSEA, stage-specific embryonic antigen; TIC, tumor-infiltrating immune cells

As shown in Fig. 2a, Kaplan-Meier survival curves showed that a high expression of SSEA-4 in cancer cells was a significant predictive factor for BCR-free survival after RP (*P* = 0.002). Similar significant relationships were also observed for SSEA-4-positive TICs (*P* = 0.007; Fig. 2b). In addition, combination of SSEA-4 expression in cancer cells and the presence of SSEA-4-positive TICs was closely associated with BCR-free survival (*P* < 0.001; Fig. 2c). Moreover, such significant prognostic roles of these SSEA-4-related parameters for BCR were confirmed by univariate Cox proportional hazard analyses (Table 3).

In contrast multivariate analysis models including the GS and pT stage showed that neither high SSEA-4 cancer cell expression or the presence of SSEA-4-positive TICs was as independent predictors of BCR-free survival (HR = 1.90, *P* = 0.095 and HR = 1.76, *P* = 0.138, respectively; Table 3). In addition, patterns characterized by either high SSEA-4 expression or SSEA-4-positive TIC presence were not significant predictors of BCR (HR = 2.25, *P* = 0.063; Table 3). However, combination of high SSEA-4 expression in cancer cells and the presence of SSEA-4-positive TICs was independently associated with BCR (HR = 2.89, 95% CI = 1.05–7.96, *P* = 0.040; Table 3).

### Correlation with cancer cell proliferation and apoptosis

**Fig. 3:**
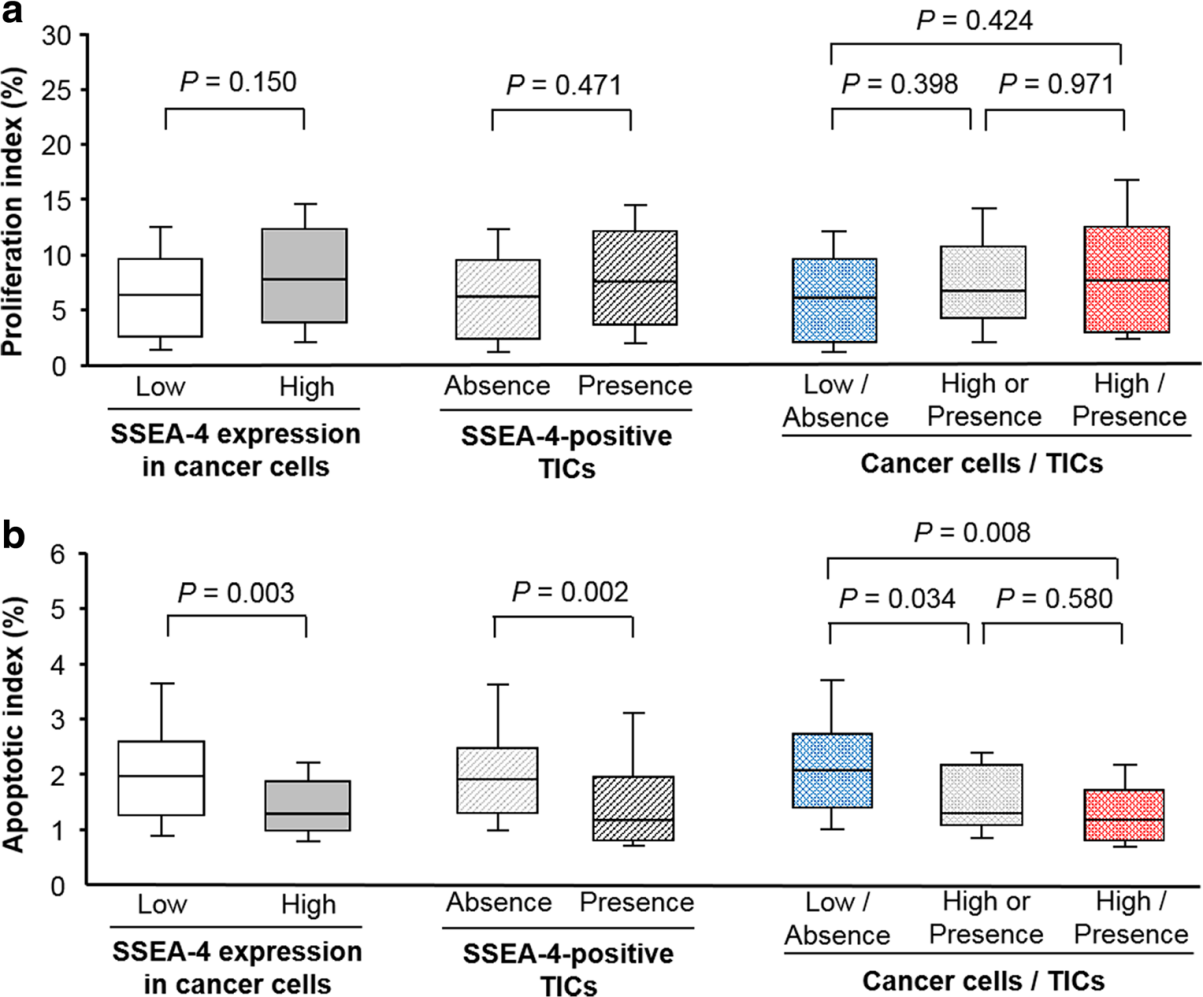
Correlation with cancer cell proliferation (**a**). SSEA-4 expression in cancer cells, presence of -SSEA-5 positive TICs, and their combination were not significantly correlated with the PI in prostate cancer patients. In contrast, the presence of SSEA-4 expression in cancer cells and SSEA-4-positive TICs was negatively associated with the AI (**b**). The AI in specimens with a high SSEA-4 expression in cancer cells and the presence of SSEA-4-positive TICs was significantly lower than that in those with negative SSEA-4 expression and the absence of SSEA-4-positive TICs. SSEA, stage-specific embryonic antigen; TIC, tumor-infiltrating immune cells; PI, proliferation index; AI, apoptotic index

As shown in Fig. 3a, there were no significant differences (*P* = 0.150) in the PI between the specimens with a high SSEA-4 expression (7.7 / 3.8–12.2) and those with a low expression (6.3 / 2.5–9.6). Similarly, the PI in specimens with SSEA-4-positive TICs (7.3 / 3.4–10.8) was similar to that in specimens without them (6.3 / 2.5–10.3; *P* = 0.471). When the relationships between the PI and pattern of SSEA-4 expression in PC cells and presence of SSEA-4-positive TICs were analyzed, significant differences were not detected (Fig. 3a).

In contrast, the AI in the specimens with a high SSEA-4 expression in the PC cells (1.3 / 1.0–1.9) was significantly lower (*P* = 0.003) than that in specimens with a low SSEA-4 expression (2.0 / 1.3–2.6) (Fig. 3b). Similarly, the AI in specimens with SSEA-4-positive TICs (1.2 / 0.8–2.0) was significantly (*P* = 0.002) lower than that in those without -SSEA-4-positive TICs (2.0 / 1.3–2.5) (Fig. 3a). In addition, as shown in Fig. 3a, the AI in specimens with a pattern of high SSEA-4 expression in PC cells and the presence of SSEA-4-positive TICs (1.2 / 0.8–1.7) was significantly lower (*P* = 0.034) than that in the case of low expression and the absence of positive TICs (2.1 / 1.4–2.8).

Based on these results, we investigated the independent role of SSEA-4-related parameters in PC cell apoptosis using multivariate analysis models including the GS and pT stage (Table 4). As a result, neither SSEA-4 expression in cancer cells or the presence of SSEA-4-positve TICs was not an independent predictor of AI (OR = 0.47, *P* = 0.115 and OR = 0.40, *P* = 0.060, respectively). On the contrary, combination of high SSEA-4 expression and the presence of SSEA-4-positive TICs was independently associated with the AI (OR = 0.21, 95% CI = 0.05–0.88, *P* = 0.032).

**Table 4:** Multivariate analyses of SSEA-4-related parameters for apoptotic index

Variables	Odds ratio	95% CI	*P* value
High SSEA-4 expression in cancer cells	0.47	0.18–1.20	0.115
Presence of SSEA-4-positive TICs	0.40	0.16–10.4	0.060
Pattern of cancer cells and TICs			
One of them; high or presence	0.59	0.23–1.50	0.265
High and Presence	0.21	0.05–0.88	0.032

Adjusted by pT stage and Gleason score

CI confidential intervals, TICs tumor-infiltrating immune cells

## Discussion

Several investigators have shown that SSEA-4 plays an important role in malignant characteristics of PC, such as regenerative potential and the loss of epithelial phenotype [10, 21, 22]. However, no study has reported on the relationships between SSEA-4 expression in cancer cells and the clinicopathological features of PC patients. The present study, for the first time, demonstrated that SSEA-4 expression in cancer cells was positively associated with the GS and T, N, and M stage in PC patients.

It was of note that in addition to cancer cells, SSEA-4 was also detected in the MSCs of PC tissues, and the presence of such SSEA-4-positive MSCs was associated with the GS, pT stage, and lymph node status in 99 patients treated with prostatectomy [10]. However, PC cells were not shown in their report whereas SSEA-4-positive MSCs were demonstrated. In contrast, the present study showed SSEA-4 expression in prostate cancer cells and the presence of SSEA-4-TICs was significantly associated with pT stage, but not the GS. Although it remains to be determined whether TICs are the same as MSCs, fibroblast-like mesenchymal stem cells stained by mAb MC813–70 [10] is morphologically different from round-shaped tumor-infiltrating immune cells (TICs) stained by RM1 in our paper. This discrepancy can be attributed to the differences of the monoclonal antibodies used, that is, the difference of the IHC results is thought to be derived from the difference of the epitopes, which the two mAbs recognize.

In addition to the correlations with clinicopathological features, high SSEA-4 expression was reported to be associated with worse prognoses and shorter survival in various cancer patients. For example, in lung cancer, patients with positive SSEA-4 expression in the cancer cells showed a 6.0-fold increased risk of relapse and a 4.2-fold increased risk of disease-related mortality [23]. These results support our results that a high SSEA-4 expression in cancer cells is predictive of a high BCR rate after RP. With regard to expression of SSEA-4 in MSCs of PC tissues, the frequency of BCR in patients with a positive SSEA-4 expression (81.8%) was significantly higher (*P* = 0.026) than that in the case of negative SSEA-4 expression (59.1%) [10]. Our results also demonstrated similar findings, that is, co-expression of higher SSEA-4 expression and presence of TICs was an independent predictor for BCR. It was speculated that stromal cells in PC tissues was speculated to play an important role in prognoses after RP.

In terms of the prognostic role of SSEA-4 in patients with PC, it is to be noted that pathological stage and the GS are well-known predictors of BCR [24–26]. Based on these facts, there is a possibility that pathological factors may affect the prognostic roles of SSEA-4. Therefore, in the multivariate model, we analyzed SSEA-4-relative parameters (expression of cancer cells and positively stained TICs) and pathological features, such as pT stage and the GS. Interestingly, combination of high SSEA-4 expression in cancer cells and the presence of SSEA-4-positive TICs was positively associated with a shorter BCR-free survival period in such patients, independently of the pathological features. Based on these results, we emphasize that co-expression of SSEA-4 on cancer cell and TICs is important to predict the BCR after RP.

Our multi-variate analysis models showed that combination of a high SSEA-4 expression in cancer cells and the presence of SSEA-4-positive TICs were significantly associated with higher invasive potential and lower apoptotic function in PC tissues. With regard to the relationships between SSEA-4 expression in cancer cells and invasion, a previous study reported the presence of SSEA-4-modulated cell adhesion and migration via interactions with integrin, F-actin, and signaling molecules in various solid tumor cells including PC cells [22]. On the other hand, a previous report demonstrated that androgen deprivation therapy led to a significant promotion of apoptosis in LNCaP cells, and this pro-apoptotic change was reversed by treatment with conditioned medium from the MSCs identified by SSEA-4 [10]. In short, SSEA-4-positive TICs may act as inhibitors of apoptosis in PC cells. These findings suggest that the co-existence of high SSEA-4 expression in cancer cells and SSEA-4-positive TICs is closely associated with worse prognoses via the suppression of apoptotic function in PC patients.

In this study, we used mAb RM1 to detect SSEA-4; this antibody is specific to SSEA-4 and does not react to GM1b or GD1a [13, 14]. Importantly, mAb RM1 is speculated to have specific characteristics similar to another mAb MC813–70. However, the two monoclonal antibodies (mAb), MC813–70 and RM1, react to different epitopes of SSEA-4. It has been shown that mAb MC813–70 reacts to sialyl T (NeuAc-Gal -GalNAc), while mAb RM1 reacts to the structure of at least 5 sugars (NeuAc-Gal-GalNAc-Gal-Gal) due to its specificity to SSEA-4. It has been shown that gangliosides interact with the other molecules [27]. Based on the facts regarding these two antibodies described above, we propose the following explanation as to why mAb RM1 was our preferred antibody to use in this study. It is probable that SSEA-4 interacts with specific molecule(s) in high-grade prostate cancer. The epitope of SSEA-4 which mAb MC813–70 recognizes may be masked through interaction with the other molecules present in prostate cancer. Thus, the access of mAb MC813–70 to its designated epitope may be limited. On the other hand, the epitope of mAb RM1 may be exposed even in the presence of interaction with other molecules, and therefore access to the epitope is not limited. In addition, RM1 recognizes upwards of five sugars, while MC813–70 recognizes only three. Therefore, the binding force of RM1, which is thought to be larger than MC813–70, could affect its capability of detecting SSEA-4.

A major limitation of this study is the relatively small sample size. Nevertheless, we demonstrated the co-existence of high SSEA-4 expression in cancer cells and presence of SSEA-4-positive TICs are important for the malignant aggressiveness in PC. Another limitation is that the evaluation of SSEA-4-positive TICs could only be performed in RP tissues, as the cancer stromal areas in the biopsy specimens were small. Conversely, the bias of evaluation on TICs in human PC tissues was minimal. Moreover, it is difficult to identify the types and characteristics of SSEA-4-positive TICs by our study design. Furthermore, to clarify the types of immune cells used by various cell markers, such as CD11c, CD45, CD34, CD4, and CD8, further studies must focus on the pathological roles of the immune system in PC tissues. On the other hand, previous reports found that tumor-derived gangliosides can bind T cells, and can then change the activity of the immune system in renal cell carcinoma [28]. Thus, there is the possibility that SSEA-4-positive TICs might be lymphocytes, including T cells. However, unfortunately, conclusive evidence was not possible with our current study design. Therefore, we used the term “TICs”, and not tumor-infiltering lymphocytes (TILs) in the present study, although the term “TILs” is commonly used to discuss immune functions in cancer tissues.

Interestingly, SSEA-4 has been reported as a marker of chemotherapeutic-resistance in certain subpopulations of patients with breast cancer [7]. However, the relationship between SSEA-4 expression and androgen-dependency or therapeutic resistance in PC has not yet been elucidated. In *in vitro* studies, we obtained data that SSEA-4 is detected in androgen-independent PC cell lines (PC-3 and DU145 cells), but not in androgen-dependent cell lines (LNCaP cells), by thin layer chromatography immunostaining (data not shown). Therefore, we speculate that responsiveness with respect to androgens might be decreased by SSEA-4 expression in PC cells. Based on these facts, we emphasize the importance of further detailed investigations on the clinical significance and pathological role at the molecular level of SSEA-4 in hormone-resistant PC.

In conclusion, the SSEA-4 expression in PC tissues was significantly higher than that in the non-tumoral tissues. In PC tissues, SSEA-4 expression in the cancer cells was positively associated with the GS and TNM stage. Multivariate analyses demonstrated that combination of high SSEA-4 expression and the presence of SSEA-4-positive TICs was an independent predictor of higher invasive potential, shorter BCR-free survival and suppression of apoptosis.
